# The Ministry of Health and the Teaching Hospitals

**Published:** 1918-11-23

**Authors:** E. W. Morris

**Affiliations:** House Governor, London Hospital.


### THE MINISTRY OF HEALTH AND THE TEACHING HOSPITALS.

By E. W. MORRIS, House Governor, London Hospital.

The Bill to establish a Ministry of Health is now on sale, and we know what will be the duties of the new Minister. Very shortly these duties are as follows:—He shall take such steps as may be desirable to secure the effective carrying out and co-ordination of measures conducive to the health of the people. This is to include measures for the prevention as well as the cure of diseases. He is responsible for the treatment of both physical and mental defects; he is to collect statistics relating to diseases, and it is to be his duty to supervise the training of persons engaged in Health Services as being one of the "measures conducive to the health of the people."

All the powers and duties of the present Local Government Board are to be transferred to the new Minister, and all the powers and duties of the Insurance Commissioners also. He is also to take over the powers of the Board of Education with respect to the health of expectant mothers and nursing mothers, and of young children. The powers of the Privy Council with regard to midwives also now pass to the new Minister. He is to be responsible for supervising the administration of that part of the Children's Act of 1908 which relates to infant life protection.

Great as these powers and duties are, more will be added to him from time to time. For instance, he may become responsible for the medical inspection and treatment of children and young persons; the powers and duties of the Minister of Pensions may soon become his, so far as the health of disabled and discharged officers and men is concerned. The care of the mentally afflicted and the administration of the Lunacy Acts will be his duty. To help him to carry out these great responsibilities Consultative Councils will be established to advise him in all matters relating to the health of the people.

In the centuries during which the great voluntary hospitals have stood watch and ward over the health of the people nothing has happened so likely to upset the even tenor of the hospital way as this Bill. We do not know whether the new Minister intends at once to interfere with present hospital methods and hospital routine, but it certainly gives him the authority to interfere. And when one notes that he is to be responsible for the "co-ordination of measures conducive to the health of the people," it is not very likely that he will close his eyes to the hospital's lack of co-ordination with everything and everybody outside its own walls. If he is to be responsible for the prevention of disease, as well as its cure, as he is, he is likely to have something to say about our laboratories, about a paid staff of whole-time workers, and about the earnest study of disease and its causes, such as cannot possibly be undertaken by a staff however eminent "whose centre of gravity is in Harley Street and not in the hospital itself." And finally, as he is responsible "for the training of persons engaged in Health Services," and as that training can only be had at our hospitals, he will have a perfect right to look into our system of medical education to see that it is providing him with the men he requires; and the women too, for surely the training of nurses will come under his jurisdiction.

To be quite frank, even if there had been no Ministry of Health at all, the time had come for us to overhaul our hospital system, and, if need be, to alter it. And I venture to say that one of the many things we have learned in the Great War is that there is a good deal in our present hospital system that cannot be defended, and must be altered.

I am more familiar with the hospitals to which medical schools are attached, and I can speak of them only with any intimate knowledge.

The present medical student is the direct descendant of the apothecary's and surgeon's apprentice of olden time. The medical schools began by the various members of the surgical staff asking the hospital board's permission to bring their apprentices down with them to the hospital to see their practice and to learn from it. At first these apprentices acknowledged no one's authority but their own chief's; they took no orders from any other chief, still less would they recognise the governing body of the hospital as having any jurisdiction over them. Later, the apprenticeship system died out, and medical students joined the hospital and were under the control of the hospital authorities. Their fees were pooled and shared amongst all the staff who taught. This system has continued in spite of obvious disadvantages. The disadvantages most apparent were that the staff taught as individuals, each as a separate well of information to his own particular set of students; he would speak of *his* theories, his experience, his method of doing this or that, and it was not an unknown thing for students to find that one member of the staff would contradict and belittle the methods of treatment of another. The fault was that the staff worked as units and not as a company of men imparting knowledge, for medicine and surgery are too big to be taught by any physician or surgeon. Another bad feature was that students were crammed for examinations instead of being properly and reasonably taught to think for themselves. They were not educated. This system could not have gone on for much longer, and the introduction of men into hospital life, men who were not on the honorary staff, but who were paid a fixed salary for their whole time—the laboratory men—the bacteriologist, the radiographer, the physiological chemist, the pathologist—the introduction of this type of men hurried the coming of the downfall of the old system. Nearly all the discoveries leading to the prevention of disease came

**Health Ministry and Teaching Hospitals**—(*cont.*)

from these whole-time men; their methods and discoveries were used by, but not created by the honorary staff, who, from the very nature of their appointments, from the comparatively short time they gave to the hospital, could not by any possibility carry out deep and long-sustained research work. It was not a question entirely of clinical methods *versus* laboratory and scientific methods; it was a question of whole-time work—all day and every day—*versus* bi-weekly visits of, say, two hours' duration. It was not the fault of the honorary staff. It was the fault of the honorary system —or perhaps, to be more accurate, the honorary system, which once did good work, splendid work, could no longer do what was necessary for the investigation and destruction of disease. There is no room for it now—or cannot be room for it much longer.

The staff themselves would not deny that it is an absolute necessity for the man who intends to practise in "Harley Street" to hold an appointment on some hospital's staff. The man who wished eventually to come on the staff would take the appointment of medical or surgical registrar, or become a demonstrator of something or other at the Medical College. He was paid in cash the wages of a carman, but he was also paid by promissory note—he would certainly some day be "on the staff." But of the thousands of young fellows who have spent four years in France, hundreds have become operators as expert as the staff of the hospitals from which they came. These men are not coming back to be paid by promissory notes. They are coming back to demand a post in their hospital—a full-time post, if you like, but a post that is well paid in cash and not by promises. Further, these same young men, who have been operating from morning to night for years, are going to seal the doom of the hundred-guinea appendicectomy. That day is past.

With all these conflicting interests and new ideals before one, what is the line that the new Minister will be likely to follow? I think he is likely to group the hospitals of the country into grades. All will become preventive as well as curative in their aims, and will be well equipped. But the hospitals with schools will have his special attention. In them are made the men who are to fill all the medical posts of his great Service, and one of his duties is to be "the training of persons engaged in Health Services." He will make these hospitals centres for the accurate observation of disease, and centres for the education of the future medical officer. The staffs of these hospitals will work in teams and not as individuals, and there will be clinics for different branches of medicine—the neurological, the genito-urinary, skin, heart, gynæcological, etc. Each unit will be run by a team of whole-time men, and well-paid men. I do not mean that such men are to fill such whole-time posts for the whole of their lives. But every man who aspires to being a sound doctor should spend some years of his life in such appointments, and especially if he ever claims to have the superior knowledge that will lead to his being "consulted." I venture to quote a paragraph from a paper written nearly two years ago on this point:—

> As has already been recommended, the hospitals should be staffed with men who would be willing to give their whole time, say for five years, to the work of the hospital for a fixed salary. Then let such a man gradually have his hospital time reduced, as he gets wiser and wiser, and his hospital salary proportionately reduced, thereby enabling him gradually to slide into private practice. New men would come on as "whole-timers," they, too, gradually working up to reduced salary and reduced hours of hospital duty. Thus many more men would reap the great educational advantage of being on a hospital staff for a period—not an honorary staff, but a well-paid staff. The pay to be based on the number of days per week the man gave to hospital; thus, if he were paid £200 a year for each whole day per week he gave, a man who has worked five days in the week would get £1,000 a year—to be proportionately reduced at the rate of £200 a year for each day he did not go to hospital. Thus even poor men would have an opportunity of becoming consultants, men who were worthy of being consulted.

There should be places in these teams for general practitioners to fill. The gain to the hospital and to the general practitioner would be mutual. The practitioner would be brought up to date in the latest developments of preventive and curative treatment, and the hospital would gain valuable information about the beginnings of disease, which it loses at present. The admission of patients from a curative point of view will not be more important than their admission from the point of view of investigation. Such purely curative work will be done at the infirmaries.

The course of training for students should become of university standard. The student should be taught, not to memorise facts, but to observe, record, weigh and balance possibilities, draw conclusions. The passing of an examination should certainly *not* be the motive of his industry; no student should work for an examination; that he has to be examined for his work is a necessary evil.

And, finally, what applies to the medical student will also apply to the nurse, for she, too, will fill an important place in this great Ministry, and her training and career should be brought under its protection.

